# Health and sanitary status in 1970 of Tubu nomads dwelling in Northeastern Niger

**DOI:** 10.1186/2054-9369-1-25

**Published:** 2014-12-02

**Authors:** Jean-François Magnaval, Christian Oosterbosch, Michel Mandl, MABN group

**Affiliations:** Department of Medical Parasitology, Faculty of Medicine-Purpan, 37 allees Jules-Guesde, Toulouse, France; CNRS UMR 5288, Paul-Sabatier University, Toulouse, France; Les Nouveaux Généralistes, Liège, Belgium; Lieutenant-General (retired), Belgian Air Force, Leefdaal, Belgium

**Keywords:** Saharian nomadic tribes, Tubu people, Health status, Sahara, Niger, Djado, Seguedine, Mission Anthropologique Belge au Niger

## Abstract

**Background:**

The Tubu are nomadic people who live in remote parts of the central Sahara, primarily in the Tibesti massif (Chad), and in both Northeastern Niger and Southern Libya. No data about the Tubu’s health and sanitary status are currently available.

**Methods:**

In 1970, the “Mission Anthropologique Belge au Niger” (MABN) investigated a Tubu tribe named Broaya that lived on the northeastern rim of the Tenere desert. One hundred and fifty-one adult volunteers were investigated. The environmental fauna of medical importance was also studied.

**Results:**

Albeit 43 year-old, these results have not been previously published. The estimated age of death for fathers was approximately 56 years, and that for mothers was 60 years. The overall perinatal mortality rate was 232%, the overall infant mortality rate was 153%, and the overall child mortality rate was 99%. The physical examination found 6 cases of blindness (4.0%). Five subjects presented with an elevated blood pressure (3.3%), and 5 (3.3%) displayed an abnormal thoracic auscultation evocative of tuberculosis or of an acute lung infection. In the field, no blood-fluke eggs were found in the urine samples. The blood thin films and stool samples were preserved then subsequently examined in Toulouse. The search for blood parasites was negative. Three subjects (2%) passed *E. histolytica/E. dispar* cysts in stools, 16 (10.6%) were parasitized with *Giardia sp.* and 4 (2.65%) were parasitized with *Hymenolepis nana*. Two specimens of scorpions captured in the camp were subsequently identified as belonging to the harmful genus *Androctonus* or *Leiurus*. An investigation into the freshwater fauna was conducted in the marshy ponds surrounding the ghost city of Djado, and no intermediate snail hosts for schistosomiasis haematobium were found. Larvae and nymphs, of *Anopheles hispaniola* and of *An. multicolor*, which are not efficient vectors for malaria, were collected.

**Conclusions:**

Infection-related blindness and trachoma, along with acute pulmonary infections and probably tuberculosis were the major health burden in this tribe. The harsh dry and hot climate may explain the low prevalence of soil-transmitted protozoan diseases or helminthiases.

## Background

For almost a decade, war has raged in Central Saharan countries - particularly in Mali and Niger - between government forces and northern rebel organizations, which are ethnic, namely Tuareg, or Islamist. A deep deterioration of the political and military situation in Mali led the UNO Security Council to adopt the 2085 resolution in December 2012 [1], giving way to military action in the field that included troops from the Economic Community of West African States through the so-called “African-led International Support Mission to Mali” from Chad and from France (“Opération Serval”). To date, military operations are still ongoing, and it is feared for many reasons that these operations will extend to other parts of Central Sahara. In that case, UNO troops would need to enter other environments; therefore, it appears crucial to have the largest possible amount of information concerning those other areas in order to carry out possible humanitarian interventions.

**Figure 1:**
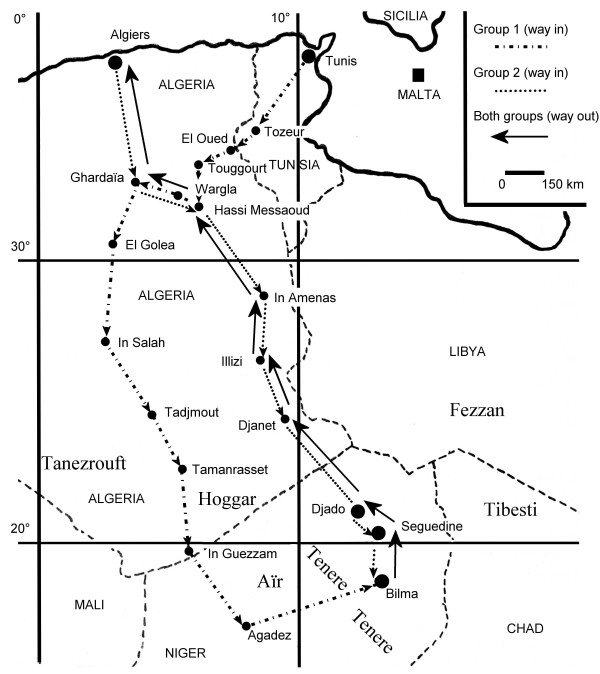
**Journeys of MABN groups in Africa.** Every reported city corresponds with a stop-over.

Northeastern Niger is the most likely candidate for an extension of military operations because this area is crossed by a trail that has potential strategic interest for rebel organizations. This route starts from the Libyan Fezzan, crosses over the Libya-Niger border and the Djado Plateau, then goes across the Tenere desert and eventually arrives in the Ar Mountains (Figure 1). The inhabitants of Northeastern Niger, which is on the fringe of the Tenere desert, are the Tubu (also known as the Teda) who remain one of the most enigmatic nomadic people in Africa [2, 3]. Tubu nomads live primarily in Northern Chad in the Tibesti massif, which is their homeland, but they also live in Southern Libya (Fezzan), Western Sudan and Northeastern Niger. Studies regarding these people are scarce and have been carried out exclusively in Chad [4–6]. Moreover, these surveys were centered on physical anthropology, ethnology and sociology, and very little is known about the health and sanitary status of Tubu tribes [7, 8]. Only one study concerning parasitology and medical entomology has been carried out in 1958 in Northern Chad under the auspices of Prohuza (French acronym for “Centre d’études et d’informations des problèmes humains dans les zones arides”) [9].

As of 2014, only three multidisciplinary missions have concerned Tenere desert and Northeastern Niger. The first ones were, by the turn of the 1960s, the famous “Berliet-Ténéré-Tchad” expeditions [10]. However, no report about health of Tubu nomads was published. The second expedition was the “Mission Anthropologique Belge au Niger” (MABN) which took place from November 1970 to the first days of January 1971. MABN intended to study all the features of Tubu people who dwelt on the Northeastern rim of Tenere desert. Anthropobiology, population genetics, and tropical medicine were the covered topics. This expedition was successful in the field, but the organization was afflicted by tragic events concerning scientific and logistic heads that elicited a dark aftermath, so only some results concerning zoology [11, 12] and Tubu physiology [13–15] have been published so far. The present article represents therefore the first public disclosure of detailed data concerning the health and sanitary status of Tubu people dwelling in Northeastern Niger.

## Methods

### Design and proceedings of MABN

MABN was created by the end of 1969 from the common goal of J.M. Wattiaux, then Head of the Department of Human Genetics at the Faculté Notre-Dame de la Paix in Namur, Belgium, of I. Vanderheyden from the Department of Endocrinology and Nutrition, Leuven University, Belgium, and of Major Avi. R. de Bruin from the Belgian Air Force. In 1969, IV (see Table 1 for initials of the names) had headed to Chad on an exploratory scientific mission entitled the “Belgian Tibesti Mission” that had demonstrated interest in further investigations regarding the Tubu people. In particular, a study of markers of genetic variation (blood groups, phenylthiocarbamide test, search for color blindness) appeared to JMW to be a possible way to understand the origin of the Tubu. Moreover, the recognized high adaption of these nomads to a very harsh environment and to a high proportion of carbohydrates in their daily food intake prompted IV to plan investigations about the Tubu’s glucose metabolism. RDB had gained a large body of field experience in the Sahara from previous humanitarian expeditions. However, by that time, the Tibesti massif had become a conflict zone between Tubu tribes and the forces of the Chad government, supported by French troops. As a consequence, the only remaining choice of the area for the mission was Northeastern Niger along the Libyan and Chad borders, where Tubu nomads reportedly dwelt.

**Table 1:** **Members and groups of the ‘Mission Anthropologique Belge au Niger”(MABN)**

Name, grade and country of members	Duty
Group 1	
M Bukowski, Gerpinnes, Belgium	Driver and mechanics
M.R. Callens, Brussels, Belgium (MRC^1^)	Nurse
J. Fairon, Tervuren, Belgium	Zoologist
Prof. P. Fuchs, Munich, Germany (PF^1^)	Ethnologist
P. Gilmont, Brussels, Belgium	Photographer
Dr. R.G. Huntsman, London, England (RGH^1^)	Hematologist
S. Jacquemart^2^, Tervuren, Belgium	Zoologist
J. Laurent, Ans, Belgium	Driver and mechanics
Dr J-F. Magnaval, Toulouse, France (JFM^1^)	Physician, parasitologist
I. Vanderheyden^2^, Leuven, Belgium (IV^1^)	Biochemist, scientific head
Group 2	
NCO A. Bosmans, Belgian Air Force, Bützweilerhof, Germany	Chief-mechanics
Major Avi. R. de Bruin^2^, Belgian Air Force, Rheindalen, Germany (RDB^1^)	Head, logistics
C. de Bruin^2^, Salmchâteau, Belgium (CDB^1^)	Nutritionist
Lt W. Kother, Belgian Air Force, Florennes, Belgium (WK^1^)	Pilot
Lt M. Mandl, Belgian Air Force, Florennes, Belgium (MM^1^)	Pilot
Dr. C. Oosterbosch, Liège, Belgium (CO^1^)	Physician
R. Serruys, Gistel, Belgium	Photographer
J. Springett, London, England (JS^1^)	Genetician
NCO G. Waeghenaere, Belgian Army, Brussels, Belgium	Driver and logistics support
Prof. J.M. Wattiaux^2^, Namur, Belgium (JMW^1^)	Genetician, scientific head
L. Welter, Belgian Air Force (reservist), Verviers, Belgium	Navigator

Military grades are as of the time of the mission. Two 4 wheel drive(WD) cars, one 4WD light truck were deployed in Group 1 and one 4WD car, one 4WD light truck and two light aircrafts in Group 2. ^1^abbrievations of names of the members which will be used in the text of this articles; ^2^Deceased.

On the scientific side, MABN gathered specialists in ethnology, hematology, nutrition, parasitology, tropical medicine and zoology from Belgium, France, Germany and United Kingdom. Militaries from Belgian Air Force and Army, accompanied by civilian volunteers, were the backbone for logistics. His Majesty Leopold II, King of Belgium, and the World Health Organization, represented by Dr. G. Lambert, head of the United Nations Research Institute For Social Development (UNRISD), were the patrons of the mission.

By the time when MABN was created (1970), such studies concerning humans did not require any ethical approval, and no ethical committees existed in the Belgian universities. However, MABN was designed to be compliant with the requirements from the first issue of the “Declaration of Helsinki” (1964).

**Figure 2:**
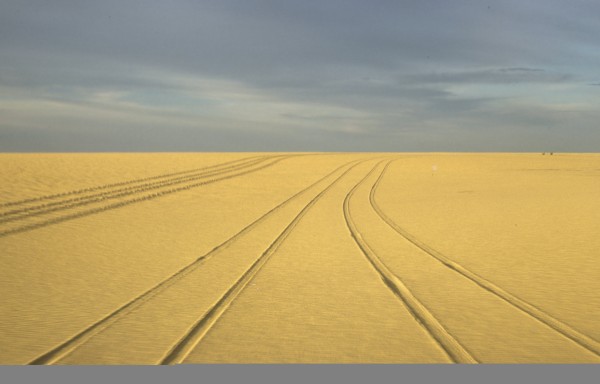
**MABN’s Group 1 on a compass route across Tenere desert.** The two tiny black spots on the right were 4WD cars of the mission.

**Figure 3:**
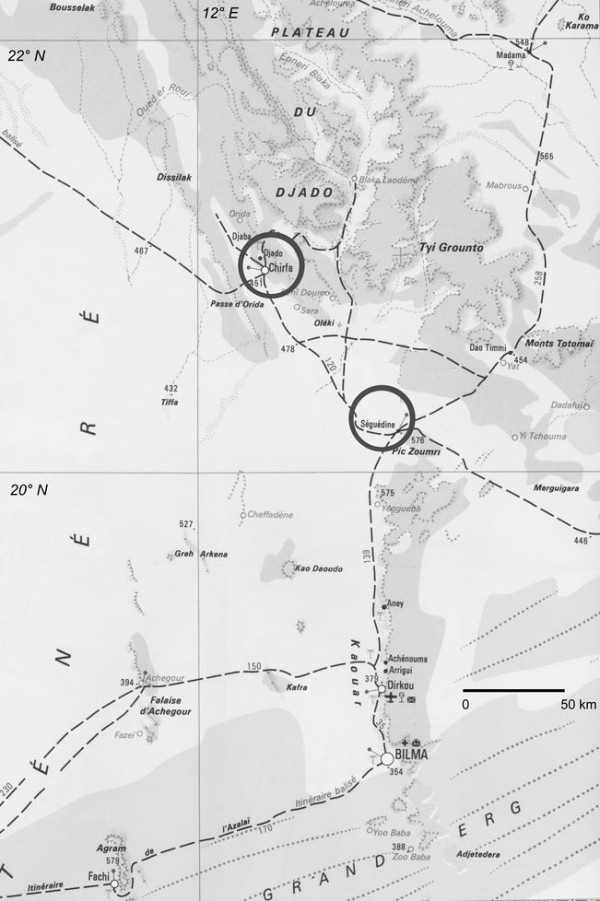
**Map of the encampments (circles) and of the area where MABN carried out the survey.** Adapted and modified from the Niger map at 1:200,000 by IGN, Paris.

In the field, MABN was split into 2 groups (Table 1) which followed different time schedules and routes (Figure 1). Briefly, one group (Group 1) entered Africa in Tunis on November 2, 1970, whereas the second group (Group 2) arrived in Algiers on November 27, 1970. The groups joined up in Bilma [latitude: 18° 41’ 7.2” (18.6853°) North; longitude: 12° 54’ 59.1” (12.9164°) East; elevation: 356 m] on December 9, 1970. Group 1 had a long journey because the mission had to purchase avgas in Agadez and carry the drums to Bilma. However, this way required crossing the Tenere desert on a 600 km-long compass route (no beacons by that time), mostly on a sandy ground (Figure 2), which made impossible the transportation of gasoline drums by MABN’s vehicles. Eventually, a pilot of the Niger Air Force accepted to convey the fuel to Bilma in a Nord-2500™ aircraft. In Bilma, local authorities informed MABN that a Tubu tribe was present at Seguedine [latitude: 20° 11’ 41.5” (20.1949°) North; longitude: 12° 57’ 39.1” (12.9609°) East; elevation: 417 m], about 165 km northbound from Bilma, and possibly at Djado [latitude: 21° 0’ 54.7” (21.0152°) North; longitude: 12° 18’ 15.4” (12.3043°) East; elevation: 449 m], about 110 km Northwest from Seguedine (Figure 3). On December 10, 1970, MABN moved to Seguedine and began field work for 6 days. On December 17, 1970, MABN went to Djado, where medical and scientific investigations were pursued until December 25, 1970. MABN left Djado on December 26, 1970, then *via* Djanet gained Algiers where embarkation was processed on January 4, 1971.

### Materials

MABN’s vehicle fleet comprised three 4WD Land-Rover™ cars, with special equipments such as 250-liter gas tanks and aviation compasses, two 4WD Unimog™ light trucks, and two Piper Cub™ light aircrafts. The ground vehicles had to carry food and gas for the period spent between Agadez and Djanet because catering and gas supplies were unavailable over the whole Tenere area and the fringes (approximately 500,000 square kilometers). The rest of the cargo comprised mainly tents and dishes, field generators, freezers (to store the blood samples), one microscope, one centrifuge, and scientific and medical items, including approximately 150 kg of various drugs. The presence of the aircrafts was necessary from the conclusions drawn from the “Missions Berliet-Ténéré-Tchad” [16]. The planes were intended to carry out the aerial recces and to guide the terrestrial vehicles if the mission had needed to search for Tubu tribes in the southern part of the Tenere desert, where long lines of high sand dunes are tangled. In fact, the aircrafts were used to locate the nomad camps along the Seguedine/Djado axis.

### Subjects

Before MABN moved out of Bilma, the heads of the mission, along with PF (an ethnologist), who spoke the Tubu language, met at Seguedine with the “Derdé” (traditional chief) of the Broaya tribe, a sub-group of the Tubu. Moreover, an aerial recce had confirmed the presence of Tubu at Djado and had found minor encampments scattered between and around both localities. The “Derdé” estimated that the aggregated size of these communities was between 750 and 800 individuals. He agreed graciously with the principles of the survey, and he verified that he would do his best to inform the families dwelling in the remote parts of the tribal territory. Subjects included in the study were therefore recruited on a voluntary basis. No payment in currency or under another form was requested by the “Derdé” or later by any Tubu volunteer.

### Anthropological and medical investigations

**Figure 4:**
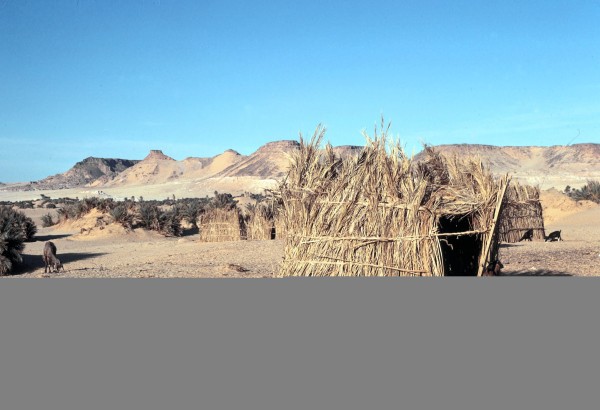
**A “buchi”, or Tubu hut, in Seguedine.**

**Figure 5:**
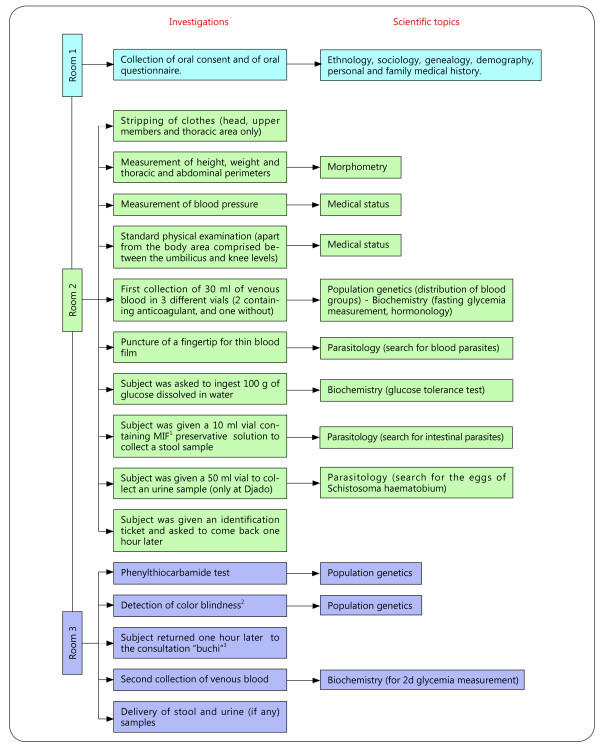
**Process of investigation for each subject.** Items in Green are investigations performed and those in Blue are scientific topics. ^1^MIF. Merthiolate-Iode-Formol. ^2^Using Ishihara’s pseudoisochromatic plates. ^3^A kind of Tubu hut.

They were carried out every morning in a large “buchi” [7], or Tubu hut (Figure 4), that was divided into 3 rooms. Every subject was received in the first room by PF (ethnologist) and a doctor (CO or JFM), along with the “Derdé” and an interpreter. Concerning the compliance with the recommendations from the 1964 version of the “Declaration of Helsinki”, only oral informed consent was collected because all of the volunteers were illiterate. Moreover, children under 14 years old (estimated age) were not accepted in the survey. Every subject answered an oral questionnaire, and personal and family information about demography, ethnology, sociology and medical history was also recorded. Investigators, “Derdé” and interpreter checked all items of the questionnaire were replied by every volunteer. Then, the volunteer entered the second room to be examined by a physician (CO or JFM), who was assisted by the MABN’s nurse (MRC). Height was measured using a rod with the subject standing on a metal plate, and weight was measured using a medical scale after stripping the thoracic area and removing any shoes. Blood pressure was checked using a sphygmomanometer and a stethoscope. In the third room, RGH and JS carried out the tests for population genetics [17]. When the subject left the “buchi”, a pilot (MM or WK) took an instant photograph that was a gift for the volunteer. A detailed list of the operations and the biological sample collection methods is displayed in Figure 5. The thin blood films were fixed in pure methanol before storage. Along with the stool samples, the blood films underwent microscopic examination in the Department of Medical Parasitology, Toulouse. Additionally, a differential count of leukocytes was then made on these slides. The urines samples were centrifuged and then microscopically examined in the field by JFM.

These nomads also received humanitarian assistance in the form of a daily medical and surgical consultation, which was held during the afternoon in the same “buchi” by one of the physicians. No doctors had visited these people since the Niger accession to independence in 1960. The “consultation ward” received anyone, regardless of whether they were a volunteer. No registration of the attendants was made, apart from 2 patients who exhibited a serious medical problem (one case of a severe lung infection combined with right-sided heart failure at Seguedine and one case of meningitis at Djado).

The environmental survey concerning parasitology and tropical medicine was carried out by JFM with the help of members of the logistics team. Various terrestrial adult arthropods were collected at Seguedine and Djado, whereas larval specimens of anopheline vectors for malaria and snail intermediate hosts for urinary schistosomiasis were collected only at Djado because no ponds or swamps existed at Seguedine. The captured specimens were stored in vials containing a 60% alcohol solution and were subsequently identified in the Department of Medical Parasitology and in the Laboratory of Zoology, Paul-Sabatier University, both in Toulouse.

## Results and discussion

First of all, it should be underlined that the study population was essentially a convenience sample rather than representative of the whole Broaya tribe, so results concerning the prevalence of observed diseases must be cautiously taken into consideration.

**Figure 6:**
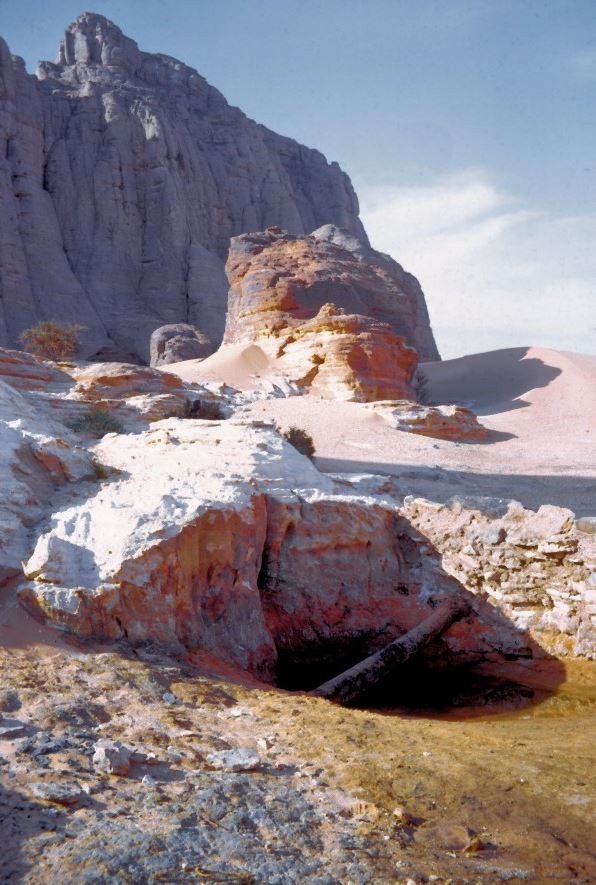
**Orida waterhole, near Djado.**

Concerning the general level of hygiene, the Tubu people demonstrated great attention to their personal care and to the cleaning of their huts and kitchen utensils. The harsh hot and dry climate represented a helpful additional factor because the “buchis” were found to be free of vermin such as cockroaches or fleas. However, the sanitary status was reduced by the presence of myriads of common flies (*Musca domestica*), by the absence of latrines and by the difficulty of having sufficient amounts of water. At Seguedine, a well in the piedmont of the hills provided moderately brackish water. At Djado, the water supply relied on a small water hole at Orida [latitude: 21° 7’ (21.1167°) North; longitude: 12° 3’ (12.05°) East; elevation: 564 m], which was 30 km Southwest of the camp (Figures 3 and 6). Obtaining clean water in this place was always an issue, and the water became muddy once the first container – called a “guerba”, or goatskin, for the Tubu, or a jerrycan for MABN - had been filled.

One hundred sixty-nine subjects, including 151 adults (54 males and 97 females) and 18 supposed teenagers (8 males and 10 females) were volunteers. It was estimated that 50 to 60 per cent of all subjects older than 17 year-old were investigated. The teenagers were medically scrutinized but were not included in the study. Table 2 displays the results from the questionnaire. Concerning demography, the Tubu did not know their precise year of birth, so no results concerning age are approximate. Conversely, the subjects remembered exactly when and how their father or mother died. Concerning family medical history, only blindness and goiters, which are obvious illnesses even for non-medical people, were included in the questionnaire. No cases of goiter were reported in the parents. The results concerning morphometry appear in Table 3, and those from the medical examinations are listed in Table 4. The Tubu were slender people and had almost no subcutaneous fat. The overall appearance of these subjects was positive, and there were not clinical signs of malnutrition,

**Table 2:** **Results of the questionnaire from the 151 adults**

Item	Value
Demography and sociology	
Average age (year, estimated)	
Males	44.6 (range: 17-80)
Females	43.5 (range: 17-75)
Average age of death (year, estimated)^1^	
Of fathers	56
Of mothers	60
Rate of violent death (accident, crime, war)^1^	
For fathers	18.3% (17/93)
For mothers	5.8% (4/69)
Average number of children per married woman (no single mother)	4.7
Death between the 6th month of pregnancy and the 3rd day post-delivery	232‰ (73/315)^2^
Death between the 4th day post-delivery and 1 year of age	153‰ (37/242)^2^
Death between 1 year of age and 10 years	99‰ (24/242)^2^
Family medical history	
Reported blindness in father^1^	8.6% (13/151)
Reported blindness in mother^1^	10.6% (16/151)
Personal medical history	
Smallpox	4.0% (6/151)
Chronic or febrile pulmonary illnesses	3.3% (5/151)
Epilepsia	1.3% (2/151)
Otorhinolaryngeal involvement	1.2% (2/151)

^1^Only one report was registered per parent and per siblings.

^2^The denominator stands for the total number of births (315) or of surviving children (242).

**Table 3:** **Results from the morphometry study of the 151 adults**

Item	Value
Average height (cm)	
Males	164.1 (range: 155–182)
Females	157.4 (range: 140-172)
Average weight (kg)	
Males	50.1 (range: 37–70)
Females	47.9 (range: 39–65)
Average thoracic perimeter at rest (cm)	
Males	79.7 (range: 64–105)
Females	76.1 (range: 62-90)
Average thoracic perimeter after breathing (cm)	
Males	84.9 (range: 66-109)
Females	80.3 (range: 64-94)
Average abdominal perimeter (cm)	
Males	69.6 (range: 51-88)
Females	67.5 (range: 54-85)

**Table 4:** **Results from the medical examination (Room #2)**

Item	Value
Average systolic blood pressure (mm Hg)	
Males	131 (range: 100–200)
Females	127 (range: 90–200)
Average diastolic blood pressure (mm Hg)	
Males	78 (range: 60–120)
Females	75 (range: 50–110)
Cases of hypertension (according to the estimated age) and accompanying symptoms	
Male (subject # 85). Transitory attacks of altered vision	0.7% (1/151)
Females. No symptom (2), chest pain on exertion (1), occipital headaches (1)	2.65% (4/151)
Possible diagnostics following physical examination	
Dentition in bad status	34% (51/151)
Trachoma	7.3% (11/151)
Blindness (sequelae from smallpox or trachoma)	4.0% (6/151)
Acute lung infection (fever, abnormal thoracic auscultation)	5.3% (8/151)
Ongoing rhinopharyngitis	4.6% (7/151)
Ongoing otitis	4.0% (6/151)
Lung tuberculosis (cachectic look, abnormal thoracic auscultation and percussion)	3.3% (5/151)
Associated digestive tuberculosis (“wooden belly” at palpation)	1.3% (2/151)
Abdominal or renal tumor or cyst (mass found by palpation)	3.3% (5/151)
Digestive amebiasis (diarrhea, painful abdominal palpation of the colon area, *E. histolytica/E.* dispar cysts in stools)	1.3% (2/151)
Hematological malignancy (enlarged spleen, lymphocytes > 80% at differential count)	1.3% (2/151)
Deaf-mute	0.7% (1/151)
Major thoracic scoliosis	0.7% (1/151)
Stroke sequelae (subject #85): chronic hypertension, Romberg’s positive, motor defect and positive Babinski sign in right lower limb	0.7% (1/151)
Tetralogy of Fallot (retarded development, cyanotic nails and lips, digital clubbing, strong systolic heart murmur)	0.7% (1/151)

A microscopic examination of the urine samples did not identify any schistosome eggs. The results concerning the thin blood films and stool samples are shown in Table 5. In the swamps at Djado, the search for mollusk intermediate hosts of *Schistosoma sp*. was negative. The examination in Toulouse of the collected specimens of entomological fauna found abundant quantities of nymphs of Ceratopogonidae (biting midges) that are vectors for human Filaria (*Mansonella sp*.) and for viruses of veterinarian importance. Concerning Culicidae, the nymphs and larvae of *Anopheles hispaniola* and *An. multicolor* were identified. Two specimens of large scorpions were determined to belong to the species *Leiurus quinquestriatus* (Seguedine) and to the genus *Androctonus sp*. (Djado). Both are considered to be very harmful.

**Table 5:** **Results of the microscopy examination of the blood thin films and stool samples**

Item	Value
Blood thin films	
Protozoan or metazoan parasites	Absent
Relative blood eosinophilia ≥ 4%	6.6% (10/151)
Stools	
Amoebozoa	
*Entamoeba histolytica/E. dispar*	2% (3/151)
*Entamoeba coli*	76% (115/151)
*Endolimax nana*	21.1% (32/151)
*Pseudolimax butschlii*	7.3% (11/151)
Trichozoa	
*Chilomastix sp.*	21.8% (33/151)
*Giardia sp.*	10.6% (16/151)
*Embadomonas sp.*	2.65% (4/151)
Helminths	
*Hymenolepis nana*	2.65% (4/151)

The Tubu’s food regimen, during the period of the survey, comprised ground seeds of the African millet (*Digitaria exilis*), fresh or dried dates, nuts of the doum palm tree (*Hyphaene thebaica*) and approximately 200 ml of goat milk daily. According to the results of the nutrition study [13], the daily caloric intake per adult subject was approximately 2,000 kcal, which came primarily from carbohydrates (ranging from 400 to 450 g, including approximately 5% from refined sugar). The amount of protein consumed ranged between 40–50 g, and 15 to 20 g of lipids consumed. Such a regimen, associated with permanent physical exertion, explains the very low prevalence of hypertension (3.3%). In comparison, the 2011 prevalence rate of hypertension was 40.2% among urban dwellers in Burkina-Faso, a country near Niger [18]. Moreover, in Africa, urbanization has been demonstrated to be a prominent risk factor for the occurrence of hypertension [19]. The morphological features of this population were close to those reported by Charpin and Coblentz for Tubu living in the Tibesti massif [4, 5]. Coblentz compared the “weight/height” ratio in the Tubu and in other Saharan nomads; he stated that the significantly lower value for the Tubu was not related to nutrition problems but indicated an adaptation to a mountainous and desert biotope.

A poor dental status was the most frequent health problem (34%) in this population. Curiously, we found poor dental statuses predominantly in subjects dwelling in Djado (50 of 51 cases), whereas this was almost absent in Seguedine (1 case). The dietary habits were similar between both communities, but the well in Seguedine had access to a water table that included lacustrian sediments which were sands and clays from the Quaternary era [20], whereas the waterhole among the Orida rocks was fed by precipitation infiltrating through the basalt structures in the Djado plateau [21]. The waters in volcanic areas may contain high concentrations of fluoride salts, which may cause dental fluorosis [22]. Likely the bacteriological quality of drinking water in both localities was poor, particularly at the Djado encampment (see above about the troubled water at Orida waterhole), and it possibly caused acute infectious diarrhea: 20.5% of the inhabitants complained of abdominal pain, liquid stools and digestive discomfort. At Djado, all of the members of MABN suffered from more or less serious attacks of traveler’s diarrhea, so prophylactic measures (daily intake of 100 mg of clioquinol) had to be taken, according to the therapeutic standards of that time. Moreover, the “death toll” rate recorded among Tubu newborns, infants and young children (Table 2) could be explained in part by a high incidence of acute digestive infections [23].

**Figure 7:**
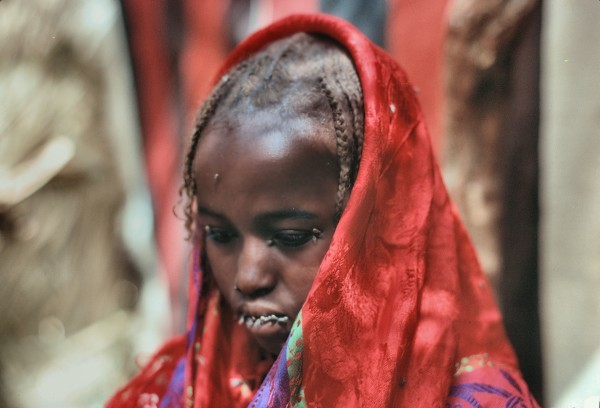
**Young Tubu girl assailed by flies.**

Infection-related blindness and trachoma were the major burdens that affected adults. The assessment of environmental parameters showed the risk factors explaining the intense transmission of *Chlamydia trachomatis* were present [24], including great poverty, less than 20 liters of water use per day, no soap supply or face washing, no latrine use and a high density of flies (Figure 7). Ranking second as major burdens were acute otorhinolaryngeal and acute lung infections, which can be explained by a poor hygiene status and by the irritating action exerted by dust or sand winds, along with the high nycthemeral variations of temperature eliciting a permanent thermal aggression: around the Achegour well, in the center of the Tenere desert, we noted that the temperature was +2°C at 04:00, +35°C (in the shade of a truck) at 13:00, and +21°C (also in the shade) at 18:00. The third scourge present in the Tubu people was tuberculosis, according to our clinical observations. In 2005, a Swiss survey that was carried out among nomad pastoralist communities in nearby Chad found a 4.6% prevalence rate of tuberculosis, also from clinical examinations [25]. This result corroborates the 3.3% rate retrieved in 1970 in the Tubu from our survey. By palpation, an abdominal mass or an enlarged kidney was detected in 3.3% of the subjects. At least one mass, which was located in the right iliac fossa (subject #110), undoubtedly had a tumor origin because it was associated with a nodular enlarged liver and a significant loss of weight. For the remaining 4 cases, hydatidosis could be another etiological hypothesis because this zoonosis is known to be highly prevalent among pastoralist populations in the East Africa Sahel [26].

The stool examination revealed that these Tubu nomads were infected by a great variety of commensal or parasite Protozoa (Table 5), a finding that can be linked to the lack of hand washing due to a deficient water supply and to the absence of latrines. However, these factors are lessened in a desert nomadic community, where people outside the family home are not in close contact and where sunlight, drought and heat have a powerful disinfecting action. Conversely, in an urban district of Niamey (Niger), the prevalence rate for *Giardia sp*. ranged from 14.9% of 322 subjects to 28.5% of 2569 subjects [27–29] compared to the 10.6% observed among the 151 Tubu nomads in the present study. Concerning intestinal helminths, the only retrieved species was *Hymenolepis nana*, a minute tapeworm whose lifecycle relies mostly upon the person-to-person transmission of embryonated eggs. The 2.65% rate was rather low compared with the 10.8% (out of 1368 subjects) found in Niamey [29] and can be explained by a lesser degree of inter-human contacts. The extreme features of the desert biotope also explain the absence of any infection due to soil-transmitted helminths (*Ascaris lumbricoides*, hookworms, and whipworms), an idiosyncrasy that was again noted in a Niamey study [27]. The very low rate of relative blood eosinophilia (Table 5) in the Tubu volunteers was a further argument for a lack of transmission for geohelminthiases.

Only one case of falciparum malaria was microscopically diagnosed and treated in Bilma, which was outside the surveyed area (Figure 3). However, in 1991, malaria was considered to be endemic in this city [30]. At Djado, *Anopheles hispaniola* and *An. multicolor* were present, but no *Plasmodium sp*. was retrieved from the examination of the blood thin films. A similar situation, which was termed in the 1920s as “anophelism without malaria” [31], had been described by Rioux in Tibesti [9]. The explanatory hypothesis reported by this author combined two factors, namely the isolated situation of the people exposed to mosquito bites and a moderate ability of certain anopheline species to transmit malaria. In fact, in 1970, the Tubu population of NE Niger only left their remote encampments once or twice a year, often traveling to Bilma or to Agadem to trade and exchange goats and camels for food (e.g., sugar, millet seeds, tea) or fabrics or tools. Moreover, *Anopheles hispaniola* and *An. multicolor* species are not considered to have much importance in the transmission of malaria in Saharan and Subsaharan Africa [32].

Based on the average estimated age of death for fathers and mothers (Table 2), we deduced that the life expectancy was 56 years for males and 60 years for females. This result is quite consistent with the 1970 data in Niger as a whole, with a life expectancy from birth of 42.6 years for males and 38.6 years for females [33].

## Conclusions

Data from a more recent book about the Tubu suggests that over the past 4 decades, no major socioeconomic changes have occurred in this society, apart from the spread of car and truck transportation [4]. In fact, the overall socioeconomic level of Niger has improved very slightly: the gross domestic product (GDP) per capita in Niger in 1970 was 97 US $ and ranked 168th in the world; in 2012, the GDP per capita was 395 US $ and ranked 205th [34, 35]. It therefore appears that the results from the MABN survey are still topical concerning the anthropology, epidemiology, and health and sanitary status of Tubu nomads in Niger. Only the malaria threat in Djado appears to be ruled out because recent satellite views have shown that the swampy areas around the ghost citadel are now dry and salty [36].

Between the beginning of the writing of this article, nine months ago, and the 2014 fall, the political and military situation has worsened in Central Sahara. French Ministry of Defence has announced that French troops will be still present in Mali, but also will be deployed in Niger and in Chad. Their stated goal will be to cut the connecting lines of Islamist organizations which use Southern Libyan Desert as a hub. French troops will be therefore in the Tubu land, so the 43 year-old data displayed in the present article may be useful for French military doctors.
